# An automated distillation method for the determination of diacetyl in beer: a comparison of analysis by AutoAnalyzer and gas chromatography

**DOI:** 10.1155/S1463924679000286

**Published:** 1979

**Authors:** J. C. Buijten, B. Holm

**Affiliations:** 1 The Department of Analytical Chemistry Stockholm University Stockholm Sweden; 2 Department of Alcohol and Drug Addiction Research Karolinska Institutet Stockholm S-104 01 Sweden


was the case with the first design. Another advantage of the procedure described is that it enables the determination of total carbon and inorganic carbon in the range 10 to 50 mg/dm$^3$ at a rate of 20 samples/h at a standard deviation of less than 1 per cent.

The described automated system is used very effectively in the authors laboratory for routine sample analysis.

ACKNOWLEDGEMENTS
This work will form part of a D.Sc. thesis in the Faculty of Mathematics and Pure Sciences at the University of Pretoria.

A special word of thanks is extended to Mr. P. J. Meaton of the Technical Services Department for original suggestions and construction of the injection units.

This paper is published with the approval of the Director of the National Institute for Water Research.

REFERENCE
[1] Van Steenderen, R. A. (1976), *Water S.A.*, **2**, 126.

### An automated distillation method for the determination of diacetyl in beer: a comparison of analysis by AutoAnalyzer and gas chromatography

**J. C. Buijten* and B. Holm**
*The Department of Analytical Chemistry, Stockholm University, Stockholm, Sweden.*

A number of colorimetric methods for the determination of diacetyl are available in which diacetyl is converted to the nickel-salt of dimethylglyoxime, West et al [1], Kielhöfer et al [2], Canales et al [3] and Brenner et al [4].

Owades et al [5] developed a micro method for the colorimetric analysis of diacetyl in beer in which diacetyl was converted to the ferro-salt of dimethylglyoxime. This method has been modified by Drews et al [6].

Another colorimetric method, developed by Gjertsen et al [7], reacts diacetyl with o-phenylenediamine to produce, 2,3-dimethylchinaxolin which absorbs at 335 nm. All the above mentioned methods are based on distillation of the beer sample.

Harrison et al [8], Arkim [9] and Bärwald [10] developed gas chromatographic methods for the determination of diacetyl in beer using a head space technique and an electron-capture detector.

The aim of the work described in this paper was to develop a fully automated method for the colorimetric determination of diacetyl in beer using a Technicon AutoAnalyzer II system, coupled to a continuous flow distillation unit. This distillation technique had previously been applied to the determination of ethanol in blood, Buijten (11).

The results from the proposed method were compared statistically with the results from parallel determinations using the gas chromatographic method.

### Methods

### Distillation unit**

The all glass distillation unit according to Buijten [11], see Figure 1, has the following dimensions:

Coil 'H': inner diameter 15 mm, length 40 cm, coil height 'I' 15 cm, coil diameter 'J' 12 cm.

Tubes 'A', 'C', 'E' and 'F': outer diameter 4 mm, inner diameter 1.7 mm. Tube 'K': outer diameter 4 mm and inner diameter 1 mm.

* Department of Alcohol and Drug Addiction Research, Karolinska Institutet, S-104 01 Stockholm, Sweden.

**Swedish Patent No. 7300722—1.

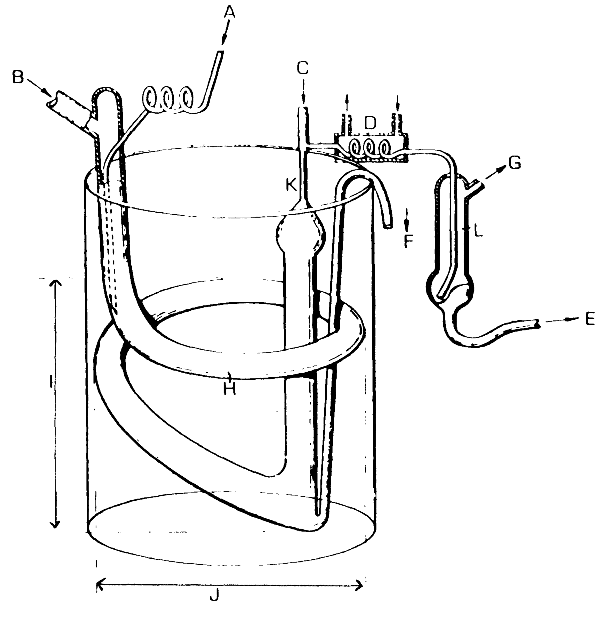

*Figure 1 Diagram of the distillation chamber.*

### The principle of the distillation unit

Volatile substances are separated from the sample by distillation in a distillation unit, which is coupled directly to a Technicon AutoAnalyzer. The samples are pumped consecutively into the distillation unit at 'A', Figure 1 and flow down into the warmed coil 'H'. Air, at approximately 2 litres per minute, is pumped in at 'B' and sweeps volatile components of the sample through to the cooling coil 'D' where they react with an absorbing reagent that has been pumped in at 'C'. The sample residue is pumped out at 'F', and excess air leaves the distillation unit at 'G'.

The absorbing reagent is pumped off at 'E' for further colorimetric analysis.

### AutoAnalyzer technique

The beer samples were degassed by placing them in an ultra-sonic bath for 15 seconds.

A schematic diagram of the manifold is given in Figure 2. The samples, (about 6 ml), were analysed at a rate of 30 per hour (sample to wash ratio, 5:1). The sample stream was mixed with a stream of 1 M potassium hydroxide solution before entering the distillation unit at 'A', where the incoming liquid was brought into contact with a gas stream (air, nitrogen or carbon dioxide) at 'B'. After the diacetyl had been distilled off, the residue of the beer sample was discarded at 'F', and the airstream containing the distilled diacetyl was mixed with the *o*-phenylenediamine reagent at 'C' and pumped through a cooling coil 'D'.

The cooled sample-reagent mixture was pumped from 'E' and into a delay coil where it was maintained in the dark at room temperature for 15 minutes. The incubated sample was then directed through a 15 mm flowcell where the absorbance of the product was measured at 340 nm and continuously recorded. Figure 3 shows a typical run of the reference standards.

### Gas chromatographic technique

Twenty ml of degassed beer was pipetted into a 50 ml bottle, which was flushed with nitrogen-gas for one minute. The bottle was stoppered and placed in a waterbath, thermostated at 40°C. After *exactly 15 minutes* a head space sample of 2.0 ml was taken (syringe warmed to 40°C) and injected into the injection port of the gas chromatograph.

A home built gas chromatograph was used for the analysis. A glass column with a length of 25 cm and an inner diameter of 2 mm, packed with TENAX-GC, 60/80 mesh (AKZO Research Laboratories), was operated at a column temperature of 90°C, injection port temperature of 110°C, and detector temperature of 110°C. The carrier gas was nitrogen at 40 ml/min, (0.8 bar). An electron-capture detector (tritium on titanium foil) was used for the determination of diacetyl. For diacetyl at 90°C: K = 7.5; N = 100 and HETP = 2.5 mm; the separation of diacetyl and 2,3-pentanedione was good, TZ = 1.0, where K = capacity ratio, N = number of theoretical plates, HETP = column length/number of theoretical plates, TZ = separation factor according to Kaiser [12]. (See Figure 4).

### Reagents

All reagents were Analytical Reagent grade.

(1) 0.25% *o*-phenylenediamine: Dissolve 0.25 g of *o*-phenylenediamine in 100 ml of 4 M hydrochloric acid. Prepare a new solution each day.

(2) 4 M hydrochloric acid: Dilute 1 part of conc. hydrochloric acid with 2 parts of distilled water.

(3) 5% v/v ethanol solution: Dilute 5 ml of ethanol (99.5%) to 100 ml with distilled water.

(4) 0.04% egg albumin — 5 v/v ethanol solution: Dissolve 40 mg of egg albumin in 100 ml of 5% v/v ethanol solution.

(5) 1 M potassium hydroxide and 0.01 M EDTA: Dissolve 56 g of potassium hydroxide and 4 g of EDTA in 1 l of distilled water.

(6) Diacetyl reference standard solutions: The purity of the diacetyl must be determined before diluting the diacetyl reference standards [13].

(a) Stock solution 'A', 1000 ppm: weight 500 mg of pure diacetyl on an analytical balance and dilute to 500 ml with albumin-ethanol solution.

(b) Stock solution 'B', 100 ppm: dilute 10.0 ml of stock 'A' to 100.0 ml with albumin-ethanol solution.

(c) Reference standard solutions: prepare from stock 'B' a series of reference standards of 0.010, 0.025, 0.050, 0.10, 0.15, 0.20, 0.25, 0.50, 0.75 and 1.00 ppm diacetyl. All dilutions should be made with albumin-ethanol solution.

### Results

### AutoAnalyzer

A standard curve was drawn using the reference standards described above. The concentration of an unknown sample was

*Figure 2 Manifold and distillation unit for the analysis of diacetyl in beer.*

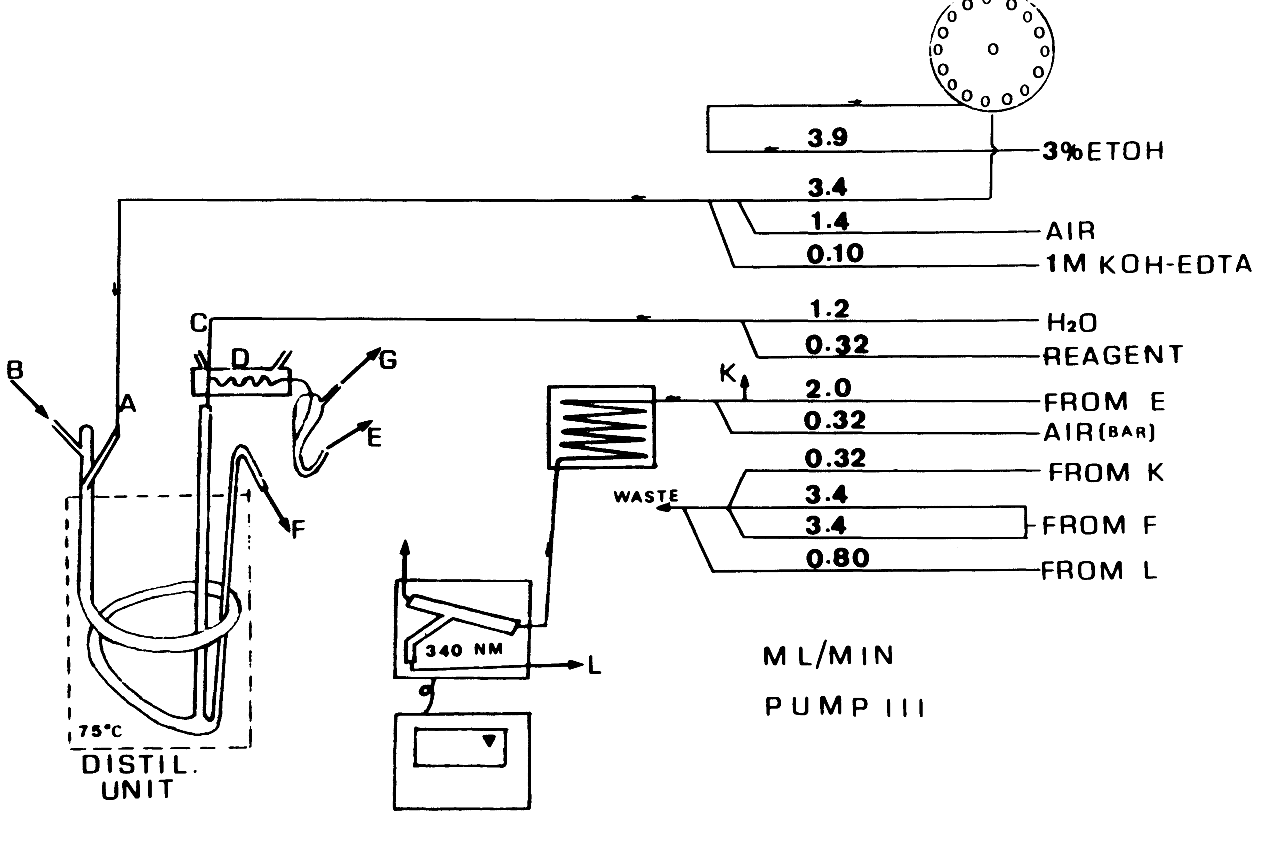

determined by comparing the peak height with that of the reference standards.

**Precision of the AutoAnalyzer method**

The precision is defined as the variability between samples, when repeating the analysis. The standard deviation ($s_d$) of the differences (AutoAnalyzer determinations, duplicate samples) was repeatable to ± 3.1%. ($s_d$ = 0.0106 ppm, $\bar{x}$ = 0.34 ppm, n = 184).

**Correlation between the two methods**

The correlation was ascertained by parallel determinations of diacetyl in beer, one set analysed according to the automated distillation method and the other set according to the gas chromatographic method. Statistical analysis of the degree of correlation between the two methods was plotted. The correlation was highly significant, R = 0.996 ± 0.016, N = 25, P = < 0.001. The regression coefficient was 0.985 ± 0.035, p > 0.05.

### Discussion

When the beer samples were compared with reference standards diluted with a 5% v/v ethanol solution, all of the results from the beer samples had to be multiplied by a factor of 1.63, in order to obtain the correct diacetyl concentration, (Table 1). When 0.05 ppm diacetyl was added to a beer sample, the recorder response increased by 8 mm, when 0.20 ppm diacetyl was added, it increased by 32 mm and when 0.50 ppm diacetyl was added to a beer sample, the recorder response increased by 80 mm (table 1-A).

After addition of the same amounts of diacetyl to a 5% v/v ethanol solution, the recorder response increased by 13, 52 and 131 mm resp. (Table 1-B); this gives a correction factor of 1.63, (13/8 = 1.63; 52/32 = 1.63; 131/80 = 1.63).

If 0.05, 0.20 and 0.50 ppm diacetyl were added to an albumin-ethanol solution, the recorder responses were the same as for the beer samples, e.g. no factor was necessary (Table 1-C). It has been reported that albumin binds diacetyl, Wainwright [13]. Five per

*Figure 3 AutoAnalyzer run of reference standards and plot demonstrating linearity.*

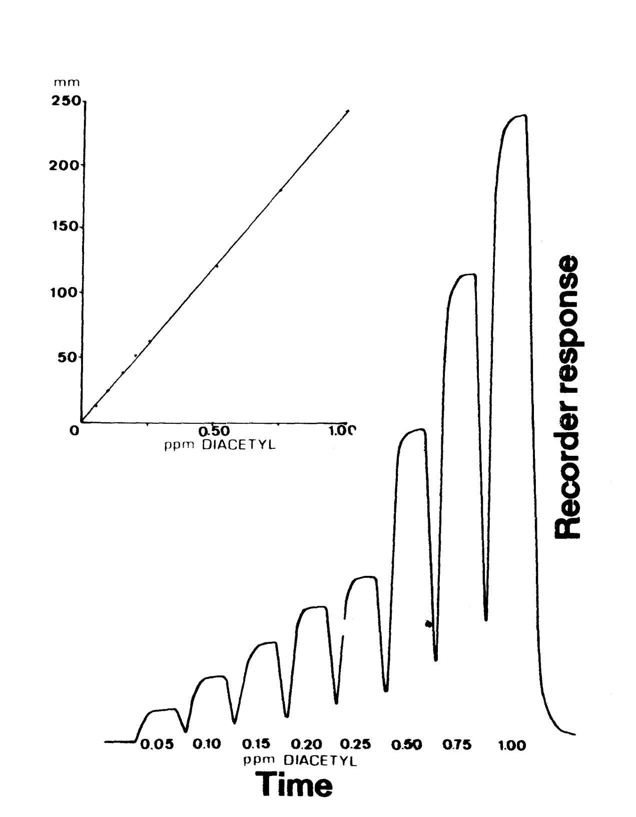

**Table I Comparison of recorder response by adding different amounts of diacetyl to beer; 5% v/v ethanol; and 5% v/v ethanol + 0.04% albumin solution**

| Sample ppm diacetyl added | Recorder response in mm: 0 | 0.05 | 0.10 | 0.20 | 0.30 | 0.50 | |
|---|---|---|---|---|---|---|---|
| Beer | 10 | 18 | 26 | 42 | 58 | 90 | A |
| 5% v/v ethanol | 0 | 13 | 26 | 52 | 78 | 131 | B |
| 5% v/v ethanol + 0.04% albumin | 0 | 8 | 16 | 32 | 48 | 80 | C |

cent v/v ethanol is generally found in most beers. Neither the amount of albumin (0.04% is an excess), nor the amount of ethanol in the albumin-ethanol solution are critical. As an alternative to the 0.04% egg albumin solution a 0.10% solution of bovine serum albumin (fraction V, Sigma) in 5% v/v ethanol can be used.

The distillation step is the most critical stage in the determination of diacetyl in beer. Prolonged distillation of the beer sample and too high a distillation temperature will give false, high diacetyl concentrations due to the conversion of α-acetolactate to diacetyl at pH > 6, Wainwright [13].

The residence time of the sample in the distillation unit described here was about *10 seconds* and the distillation temperature was 75°C. No differences in diacetyl concentration were observed when either air, nitrogen or carbon dioxide were used

*Figure 4 A typical head space chromatogram of a beer sample.*

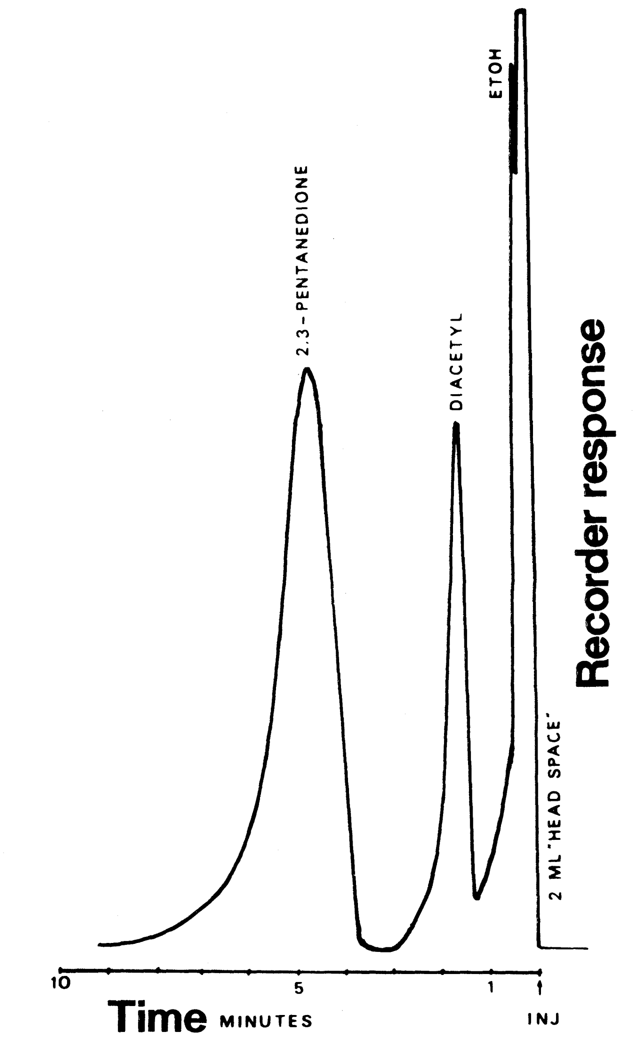

for the gas stream in the distillation unit.

Coagulation of the beer samples, especially when running a large number of samples, can arise in the distillation unit. Coagulation interferes with the smooth flow of sample through the distillation unit and gives erratic results. Coagulation was prevented by mixing the sample with a 1 M potassium hydroxide solution (Figure 2) before pumping it into the distillation unit. The pH of the beer sample then varied between 6 and 7.

ACKNOWLEDGEMENTS
We are obliged to Technicon Scandinavia AB for all the help with the AutoAnalyzer II system and to Ing Thelemann (Warby Bryggeriet) for the beer samples.

REFERENCES

[1] West, D. B., Lautenbach, A. L. and Becker, K. *Proceedings of the American Society of Brewing Chemists,* 1952, 81.
[2] Kielhöfer, E. and Würdig, G. *Weinwissenschaft,* 1960, **15**, 135.
[3] Canales, A. M. and Martinez, N. *American Brewery,* 1962, 10.
[4] Brenner, M. W., Blick, S. R., Frenkel, G. and Siehenberg, J. *Proceedings of the European Brewery Convention,* Brussels, 1963, 233.
[5] Owades, J. L. and Jakovac, J. A. *Proceedings of the American Society of Brewing Chemists,* 1963, 22.
[6] Drews, B., Specht, H., Bärwald, G. and Trenel, G. *Monatsschrift Brauerei,* 1966, **19**, 34.
[7] Gjertsen, P., Undstrup, S. and Trolle, B. *Monatsschrift Brauerei,* 1964, **17**, 232.
[8] Harrison, G. A. *European Brewery Convention, Proceedings of the 10th Congress,* Stockholm, 1965.
[9] Arkim, A. *82nd Congress of the American Brewmasters in Toronto,* 1968.
[10] Bärwald, G. *Monatsschrift Brauerei,* 1970, **23**, 12.
[11] Buijten, J. *Blut Alkohol,* 1975, **12**, 393.
[12] Kaiser, R. E. and Rieder, R. *Chromatographia,* 1977, **10**, 455.
[13] Wainwright, T. *Journal of the Institute of Brewing,* London 1973, **79**, 451.

### Clinical laboratory evaluation of the Orion SS-20 ionized calcium analyser

**Patrick Ferreira**
*Department of Laboratory Medicine, University of Alberta Hospital, Edmonton, Alberta T6G 2B7, Canada.*

**Alan M. Bold**
*Department of Clinical Chemistry, Queen Elizabeth Medical Centre, Edgbaston, Birmingham B15 2TH, England.*

### Introduction

About 50% of the total plasma calcium exists as free calcium ions (ionized calcium, $Ca^{2+}$), and this is generally considered to be the physiologically active fraction. There is evidence to suggest that its direct measurement is of greater value than total calcium in various disorders [1,2]. Until recently techniques for measurement of ionised calcium (spectrophotometric [3-5] and potentiometric [6-12]) have been too unreliable or time-consuming for routine use in most laboratories. pH and temperature affect the binding of calcium to protein [4], and the necessity to control these variables has led to further analytical complications.

With the introduction of the Orion model SS-20 ('Space Stat'-20) this situation could change. A recent advertisement [13] for the analyser stated: 'Operation is simple: the sample is injected, a button is pushed and the result read on the digital display. Now this important blood parameter can be measured throughout the hospital — the central laboratory, in satellite laboratories, in intensive care, in pediatrics. Ionized calcium can now join the list of routine clinical tests.'

Indeed, five recent publications have essentially supported this claim [14-18], but the authors' experience has not been so favourable and some shortcomings of the instrument have been found in this evaluation that have not been previously recognized. These findings are factors which should be considered by potential users of the instrument.

### Materials and methods

### The SS-20 analyser

It is not intended to give a detailed description of the analyser, this is available from the manufacturer: Orion Research Inc., 380 Putnam Ave., Cambridge, Mass. 02139, U.S.A. The instrument is basically designed to measure $Ca^{2+}$ at 37°C in an anaerobic sample of whole blood, plasma or serum. This is performed in a three minute automated cycle in which the sample is pumped past a liquid ion-exchange type of calcium electrode (the 'sensor'). The reference electrode is Ag/AgCl through which a slow flow of 2 mol/l KCl saturated with $Ag^+$ is pumped, this then meets the sample stream at a liquid junction. A standard (1.00 mmol/l $Ca^{2+}$) is pumped past the sensor following the sample. From the difference in potentials, and using a 'slope' factor calibrated in each run using aqueous standards, the instrument automatically calculates and displays the $Ca^{2+}$ result. The reagents and standards used were as those supplied by the manufacturer, and the analyser was used in accordance with the manufacturer's instruction manual. Sensor potentials were monitored on a chart recorder throughout the study.

### Ionized calcium: spectrophotometric method

The method of Varghese [5] was used: This involved preliminary anaerobic ultrafiltration at 37°C, followed by dual-wave-length spectrophotometry of tetramethyl murexide added to the ultrafiltrate.

### Dialysable calcium method

The method of Ferreira and Bold [19] was used. This involves continuous-flow dialysis at 37°C of an anaerobic sample, with detection of calcium in the dialysate using o-cresolphthalein complexone.

pH was measured using a Radiometer BMS Mk2 blood gas analyser.

Total calcium was measured by the standard SMA-12/60 o-cresolphthalein complexone method (based on No. $AA_2$03). (Technicon Corp., Tarrytown, U.S.A.).

### Sample preparation

Venous blood was collected into heparinized polypropylene syringes, which were capped. The dilution of heparin was chosen to give a final concentration of 3-4 units/ml of whole blood. The syringes were centrifuged, nozzles uppermost, and the separated plasma was drawn off into a second set of syringes using short sleeves of plastic tubing to connect the syringe nozzles. All analyses were performed within four hours of collection.

### Control sera

Three levels of $Ca^{2+}$ were prepared by pooling hospital in-patient samples (excluding jaundiced or haemolysed sera). The 'high'

